# Early Spatiotemporal Patterns and Population Characteristics of the COVID-19 Pandemic in Southeast Asia

**DOI:** 10.3390/healthcare9091220

**Published:** 2021-09-16

**Authors:** Mingjian Zhu, Jirapat Kleepbua, Zhou Guan, Sien Ping Chew, Joanna Weihui Tan, Jian Shen, Natthjija Latthitham, Jianxiong Hu, Jia Xian Law, Lanjuan Li

**Affiliations:** 1 State Key Laboratory for Diagnosis and Treatment of Infectious Diseases, National Clinical Research Center for Infectious Diseases, Collaborative Innovation Center for Diagnosis and Treatment of Infectious Diseases, The First Affiliated Hospital, Zhejiang University School of Medicine, Hangzhou 310003, China; 11618126@zju.edu.cn (M.Z.); 12018354@zju.edu.cn (Z.G.); 21718042@zju.edu.cn (J.S.); 2 Thammasat University Hospital, Pathum Thani 12120, Thailand; kleepbua.zju@gmail.com (J.K.); natthjijal@gmail.com (N.L.); 3 School of Medicine, Shanghai Jiao Tong University, Shanghai 200025, China; chewsienping@sjtu.edu.cn; 4 Faculty of Arts and Social Sciences, National University of Singapore, Singapore 117570, Singapore; e0322819@u.nus.edu; 5 Guangdong Provincial Institute of Public Health, Guangdong Provincial Center for Disease Control and Prevention, Guangzhou 511430, China; hzeros_hu@163.com; 6 Tuanku Ja’afar Hospital, Seremban 70300, Malaysia; DocLaw811@gmail.com

**Keywords:** COVID-19, epidemic pattern, exponential growth, basic reproduction number (***R***_0_), spatio-temporal analysis, demographic risk factor, observational study, public health, Southeast Asia (SEA)

## Abstract

This observational study aims to investigate the early disease patterns of coronavirus disease 2019 (COVID-19) in Southeast Asia, consequently providing historical experience for further interventions. Data were extracted from official websites of the WHO and health authorities of relevant countries. A total of 1346 confirmed cases of COVID-19, with 217 recoveries and 18 deaths, were reported in Southeast Asia as of 16 March 2020. The basic reproductive number (***R***_0_) of COVID-19 in the region was estimated as 2.51 (95% CI:2.31 to 2.73), and there were significant geographical variations at the subregional level. Early transmission dynamics were examined with an exponential regression model: y = 0.30e^0.13x^ (*p* < 0.01, R^2^ = 0.96), which could help predict short-term incidence. Country-level disease burden was positively correlated with Human Development Index (r = 0.86, *p* < 0.01). A potential early shift in spatial diffusion patterns and a spatiotemporal cluster occurring in Malaysia and Singapore were detected. Demographic analyses of 925 confirmed cases indicated a median age of 44 years and a sex ratio (male/female) of 1.25. Age may play a significant role in both susceptibilities and outcomes. The COVID-19 situation in Southeast Asia is challenging and unevenly geographically distributed. Hence, enhanced real-time surveillance and more efficient resource allocation are urgently needed.

## 1. Introduction

An unknown infectious disease caused by severe acute respiratory syndrome coronavirus 2 (SARS-CoV-2) emerged in Wuhan, China, in December 2019 [1]. The disease was later officially named coronavirus disease 2019 (COVID-19). The World Health Organization (WHO) declared COVID-19 a pandemic on 11 March 2020 due to its rapid global spread [2]. An analysis of outbreak and global impacts of the COVID-19 have visually depicted how the disease managed to affect nearly 150 countries/territories/areas over a span of just two months [3].

The global outbreak of COVID-19 has been ongoing in Southeast Asia since 13 January 2020, making Southeast Asia the first affected region outside of China [4]. On 1 February 2020, the Philippines’ Department of Health (DOH) reported the first COVID-19 mortality outside of China [5]. Southeast Asia (SE Asia or SEA) is composed of 11 sovereign states, which are divided into three groups geographically: mainland SEA (Cambodia, Laos, Myanmar, Thailand, and Vietnam), maritime SEA (Brunei, Indonesia, Malaysia, the Philippines, and Timor-Leste), and Singapore as a junction point. As a regional unit, it not only borders China but also lies at “crossroads of the world” due to important maritime trade routes [6]. To control the pandemic, ASEAN (the Association of Southeast Asian Nations) countries generally took similar intervention measures but there were differences in the spread, burden, and medical capacities [7]. In the context of globalization, regional disease surveillance is essential because it contributes to the formulation of responses to such emerging infectious diseases [8]. Capturing the baseline transmission characteristics of novel coronavirus in specific social contexts is strategic for programming public health interventions [9].

Some previous studies conducted early epidemiological analyses of COVID-19 outbreaks in individual SEA countries [4,10,11,12], but there are no region-wide studies focused on this topic. Moreover, the effectiveness of intervention strategies implemented in the early stage still remains to be evaluated by analyzing the existing surveillance data. The purpose of this observational study is to investigate the underlying disease patterns of COVID-19 in this region, and consequently to guide the pandemic responses in the future.

## 2. Materials and Methods

### 2.1. Study Design

In this population-level observational study, we performed a retrospective analysis of COVID-19 epidemiological data from all 11 countries in Southeast Asia during the period between 13 January 2020 and 16 March 2020. Primary data sources were the official websites of the WHO and the public health authorities (such as the Ministries of Health or Centers for Disease Control) in relevant countries. We included individuals with a positive polymerase chain reaction (PCR) test for SARS-CoV-2 (*n* = 1346). An Excel spreadsheet database was created through data compilation and used for analyses. First, we illustrated the temporal and spatial distributions of the COVID-19 outbreak in Southeast Asia and further detected the potential spatiotemporal cluster(s). Then, a simple mathematical model for the growth of confirmed cases was established based on the temporal distributions and subsequently applied to the short-term trend analyses. Finally, we described the demographic characteristics of infected individuals and further identified the probable risk factors for morbidity and mortality. Additionally, the perspective of subregional comparison was adopted throughout the study to reveal internal differences.

### 2.2. Data Compilation

We closely monitored updates from press releases and situation reports on COVID-19 issued by each Southeast Asian country’s health authorities and the WHO between 13 January 2020 and 16 March 2020. Using a structured information form, our multilingual team directly and in real-time extracted epidemiological data that included daily case counts, outbreak maps, and basic demographic characteristics such as age, sex, and nationality. Additionally, data of actual case counts in the subsequent 35 days (17 March 2020–20 April 2020) were collected. We only compiled individual-level data for 925 cases that tested positive for COVID-19 since health authorities in Malaysia and Indonesia did not disclose relevant details after 13 March 2020, which resulted in a reduction in the sample size for demographic analysis. General population data at the national level (including population size, median age, and sex ratio) were extracted from World Population Prospects 2019 (United Nations, Department of Economic and Social Affairs) and the World Bank Open Data. Human Development Index (HDI) data at the national level were extracted from Human Development Report 2020 published by the United Nations Development Programme. After cross-checking, all extracted data were entered into an Excel spreadsheet database for further quantitative analysis.

### 2.3. Statistical Analysis

Descriptive statistical methods were used to analyze the spatiotemporal and population distributions of COVID-19 in Southeast Asia. Epidemic curves and semilogarithmic graphs were constructed by the report date using Microsoft Excel 2019 (Microsoft, Redwoods, WA, USA). The burden of disease was measured at the regional, subregional, and national levels by cumulative incidence (per 1,000,000 population). The spatial distribution of confirmed cases was illustrated with marked maps, using R software version 3.6.2 (R Foundation for Statistical Computing, Vienna, Austria). We also assessed the age, sex, and nationality of individuals with COVID-19 and those who died of it. Demographic data were expressed as median (interquartile range, IQR) or n (%), as appropriate. Crude recovery or fatality rates were calculated based on the reported cumulative counts.

An exponential regression model was constructed to estimate short-term incidence trends using SPSS statistical software version 26.0 (IBM Corp., Armonk, NY, USA), and then we tested the statistical significance by the analysis of variance (ANOVA) and evaluated the goodness-of-fit with the adjusted R-square. To further evaluate the modeling effect as well as the trend change, a comparative analysis was performed between the predicted counts and the actual counts for the subsequent 35 days.

Three key transmissions dynamic parameters of the early COVID-19 outbreaks were estimated for the entire region and three subregions. The exponential growth rates were obtained from the exponential regression models described above. The average doubling times were calculated using a method proposed by Galvani et al. [13]. As an epidemiological metric of viral transmissibility, the basic reproductive number (***R***_0_) refers to the average number of secondary cases generated by one primary case in a fully susceptible population [9,14]. In the study, based on the time series of COVID-19 new case counts, ***R***_0_ was estimated by the maximum likelihood (ML) method using the ***R***_0_ package in R software version 3.6.2 (R Foundation for Statistical Computing, Vienna, Austria) [15]. In the calculation process, we assumed that the generation interval of COVID-19 conformed to the Gamma distribution with a mean of 5.20 and a standard deviation of 1.72 days [16].

Using the QGIS 3.16 software (Open Source Geospatial Foundation, Beaverton, OR, USA), a bivariate choropleth map was constructed to co-present the cumulative incidence rates and the corresponding HDI values. To quantify the association between these two variables, Spearman’s correlation analysis was further implemented using SPSS statistical software version 26.0 (IBM Corp., Armonk, NY, USA). A retrospective space-time analysis based on the discrete Poisson model was performed to scan for clusters with high COVID-19 incidence rates using SaTScan software version 9.7 (Martin Kulldorff and Information Management Services Inc., Boston, MA, USA) [17]. Both of the maximum spatial clustering scale and the maximum temporal clustering scale were set to 50%. The window with the largest log-likelihood ratio (LLR) value was selected as the most likely cluster. The scanning results were visualized on a map using ArcMap software version 10.2 (ESRI Inc., Redlands, CA, USA).

Kruskal-Wallis tests and Chi-square tests were used to compare the distributions of numerical variables (age) and categorical variables (sex and nationality) among the three subregions, respectively. Paired *t*-tests and Mann-Whitney U tests were used to compare median ages and sex ratios between patients and general populations, as well as to make comparisons between deceased and surviving cases. All of the hypothesis tests were performed using SPSS statistical software version 26.0 (IBM Corp., Armonk, NY, USA), and a *p*-value < 0.05 was considered to be statistically significant.

### 2.4. Ethical Approval

Ethical approval or individual consent was not applicable.

## 3. Results

As of 16 March 2020, 1346 confirmed cases of COVID-19 were reported in Southeast Asia. Of these, 217 patients recovered and 18 patients died. The crude recovery and fatality rates were 16.1% and 1.3%, respectively.

### 3.1. Temporal Distribution and Incidence Trends

**Figure 1:**
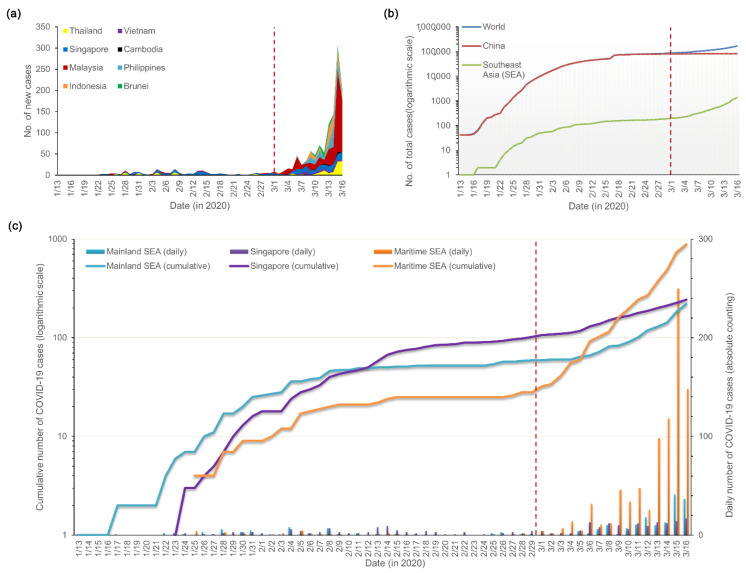
Temporal distribution of confirmed COVID-19 cases in Southeast Asia from 13 January to 16 March 2020: (**a**) Epidemic curve by date of report and country; (**b**) semi-logarithmic graph of total cases for the World, China, and Southeast Asia; (**c**) Statistical chart showing the subregional variations in COVID-19 time series. Red dashed line: watershed (on 1 March) between the first and second phases.

An epidemic curve of confirmed cases (by report date) indicated that there were two distinct phases: (1) 13 January–29 February, 2020 (first phase) and (2) 1 March–16 March, 2020 (second phase). The situations during the first phase of the outbreak were relatively mild, with only a few confirmed cases reported daily, and most were from Singapore and Thailand. However, in the second phase, the daily reported numbers of confirmed cases increased rapidly, especially in Malaysia. The highest jump in new COVID-19 infections was recorded in Malaysia on 15 March 2020, with a single-day increase of 190 new cases (Figure 1a).

A semi-logarithmic graph of cumulative cases over time revealed that the diffusion of COVID-19 in Southeast Asia significantly accelerated at the beginning of March 2020 and was higher than the global level. In contrast, the COVID-19 situation in China remained stable (Figure 1b). Geographic variation in the early growth trajectory of COVID-19 cases within the region was observed (Figure 1c). It took 31, 20, and 39 days, respectively, for mainland SEA, Singapore, and maritime SEA to reach 50 COVID-19 cases after the first confirmed case was reported. Although the date of the first confirmation report in maritime SEA was 12 days later than that in mainland SEA, the confirmed cases from maritime SEA saw a steep growth in the second phase and exceeded half of the total cases from the whole region by mid-March 2020.

During the study period, the burden of the pandemic in Southeast Asia was relatively low (2.03/million population) but large internal differences existed (Table 1). The average doubling time and basic reproduction number of the early COVID-19 outbreak in the region were estimated as 6.16 days and 2.51 (95% confidence interval:2.31 to 2.73), respectively. Among three subregions, mainland SEA had the longest doubling time (8.22 days), whereas maritime SEA had the highest basic reproductive number (4.16).

**Figure 2:**
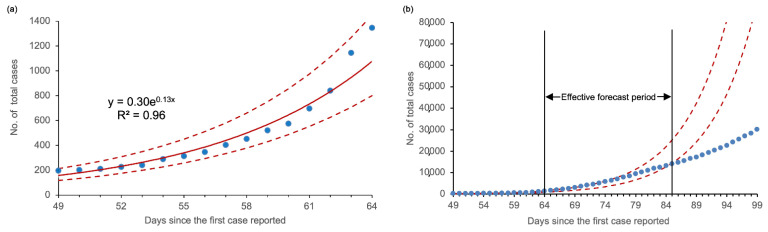
Modeling the early incidence trends of COVID-19 in Southeast Asia: (**a**) Exponential curve fitting for the growth of the cumulative number of confirmed cases since 1 March 2020; (**b**) comparative analysis of predictions vs. observations within 35 days demonstrating a 20-day effective forecast. Blue dots: actual distribution of values; red solid line: exponential regression curve; red dashed lines: upper and lower limits of 95% confidence interval.

An exponential curve was used to fit the incidence trends in the second phase. We obtained the following regression model: y = 0.30e^0.13x^ (y is the cumulative number of confirmed cases in the second phase and x is the number of days from the first reported case in Southeast Asia). ANOVA indicated that this model was statistically significant (F = 355.48, *p* < 0.01), and the adjusted R^2^ = 0.96 (Figure 2a). According to the model, the cumulative number of confirmed cases of COVID-19 in Southeast Asia was predicted to exceed 10,000 by Day 81 (2 April 2020). The incidence trends within 20 days were effectively forecast, yet since Day 85 (6 April 2020), the actual situations have gradually shifted to a lower-than-expected phase (Figure 2b).

### 3.2. Spatial Distribution and Spatiotemporal Clusters

By 16 March 2020, eight countries in Southeast Asia (except Laos, Myanmar, and Timor-Leste) reported confirmed cases of COVID-19. Malaysia (*n* = 553), Singapore (*n* = 243), and Thailand (*n* = 147) reported the highest numbers of COVID-19 infections, accounting for 70.1% of the total cases reported in Southeast Asia. Notably, Singapore had the highest number of recovered cases (*n* = 109) with a crude recovery rate of 44.9%. The most deaths occurred in the Philippines (*n* = 12) and Indonesia (*n* = 5) with crude fatality rates of 8.5% and 3.7%, respectively.

**Figure 3:**
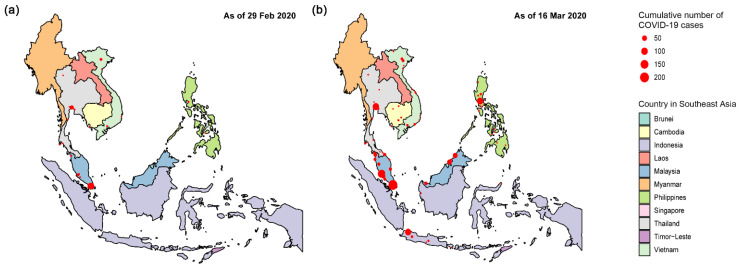
Spatial distribution of COVID-19 cases in Southeast Asia as of (**a**) 29 February and (**b**) 16 March 2020.

In the first phase, 69.3% of the confirmed COVID-19 cases were primarily concentrated in two major international metropolises (Singapore and Bangkok) (Figure 3a). Onset focus areas of COVID-19 infections expanded to other international metropolises in this region, including Manila, Kuala Lumpur, and Jakarta. The number of affected municipalities or first-level administrative units rose to 74, giving the pandemic a “cancer metastasis-like” spatial distribution, especially in the Malay Peninsula (Figure 3b).

**Figure 4:**
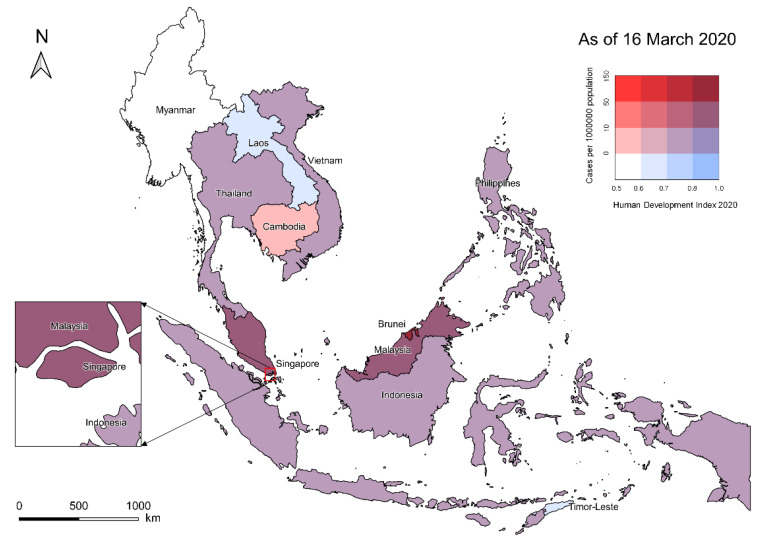
Bivariate choropleth map illustrating cumulative COVID-19 incidence rates and corresponding HDI values at the national level. The darker blue countries represent the areas with the lowest disease burden yet the highest HDI. The dark red countries represent the areas with the highest disease burden and the highest HDI.

During the first nine weeks of the COVID-19 pandemic in Southeast Asia, Brunei, Singapore, and Malaysia had the highest disease burden and their cumulative incidence rates were 124.63, 41.87, and 17.31 cases per million population, respectively. Interestingly, countries with the highest disease burden in the region were among those with the highest HDI values (Figure 4). Spearman’s correlation analysis revealed a strong positive correlation between country-level cumulative incidence rates and HDI values (r = 0.86, *p* < 0.01).

**Figure 5:**
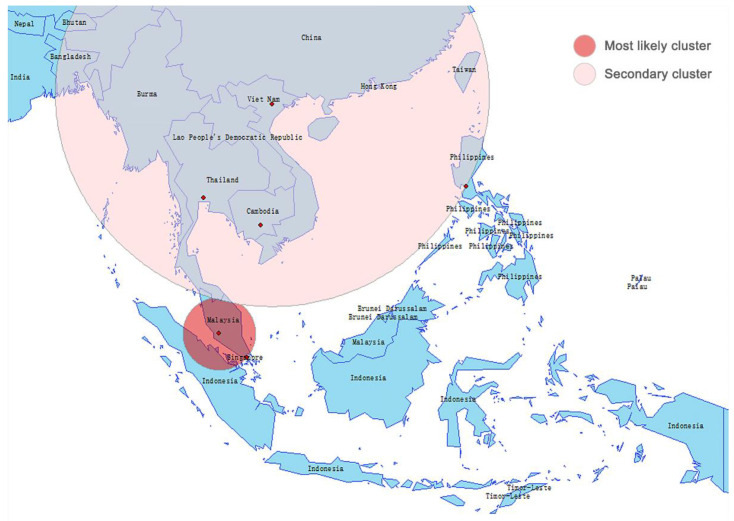
Spatial-temporal scan showing the significant aggregation effect of early COVID-19 outbreak in Southeast Asia, with the most likely cluster concentrated in the Malay Peninsula.

Two spatiotemporal clusters with statistical significance were detected, and both occurred in the second phase (Figure 5). The most likely cluster involved Malaysia and Singapore during 4 March–16 March, 2020 (RR = 72.07, LLR = 1910.08, *p* < 0.001). The secondary cluster occurred during 11 March–16 March, 2020 (RR = 4.53, LLR = 160.75, *p* < 0.001), covering a wider geographical area (Vietnam, Thailand, Cambodia and Philippines).

### 3.3. Demographic Characteristics and Risk Factors

The sample size for the demographic analysis was 925. Of these, the age of one patient from Cambodia and the sex of one patient from Indonesia were unknown because the health authorities in Cambodia and Indonesia did not publish this information. Moreover, 104 cases from Malaysia were missing values for age; these data were imputed with a stochastic simulation method based on the age distribution of confirmed cases as of 13 March 2020 issued by the Ministry of Health, Malaysia [18]. Table 2 summarizes the demographic characteristics of confirmed COVID-19 cases. At the subregional level, the median age of infected individuals in mainland SEA was the lowest (37 years), while the proportion of non-foreign nationality in maritime SEA was the highest (91.6%).

**Figure 6:**
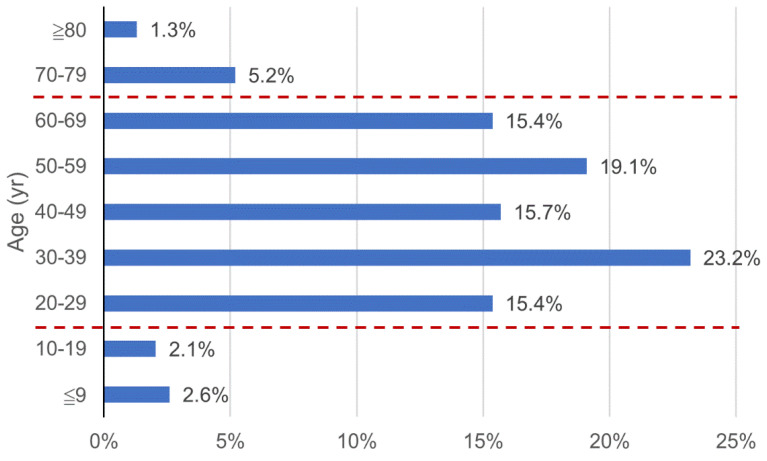
Age distribution of confirmed COVID-19 cases in Southeast Asia. Red dashed lines: upper and lower limits of the age group in which nearly 90% of cases were concentrated.

Demographic analysis revealed that COVID-19 patients were primarily aged 20–69 years (Figure 6). This age group constituted 88.8% of the total confirmed cases in Southeast Asia. The proportion of COVID-19 cases among individuals aged ≥60 years was 21.9%. The ages of individuals with COVID-19 in Southeast Asia ranged from 0.25–96 years, with a median age of 44 years. There were 514 males and 410 females, with a sex ratio of 1.25.

**Figure 7:**
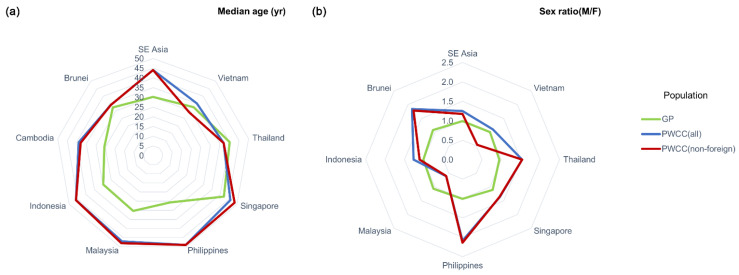
Demographic constitution of confirmed COVID-19 cases in Southeast Asia (SE Asia) in terms of (**a**) age and (**b**) sex. GP: general population; PWCC: population with confirmed COVID-19.

The median ages and sex ratios of the population with confirmed COVID-19 (PWCC) and the general population (GP) from each country are presented in Figure 7a,b, respectively. For early non-foreign infected individuals, the highest median age and sex ratio were found in the Philippines (49 years and 2.14) and the lowest were found in Vietnam (29 years and 0.54). Moreover, the median age of PWCC (non-foreign) was significantly higher than that of the corresponding GP (paired *t*-test; *p* < 0.01), whereas the sex ratio did not significantly differ between the two population groups (paired *t*-test; *p* > 0.05).

**Figure 8:**
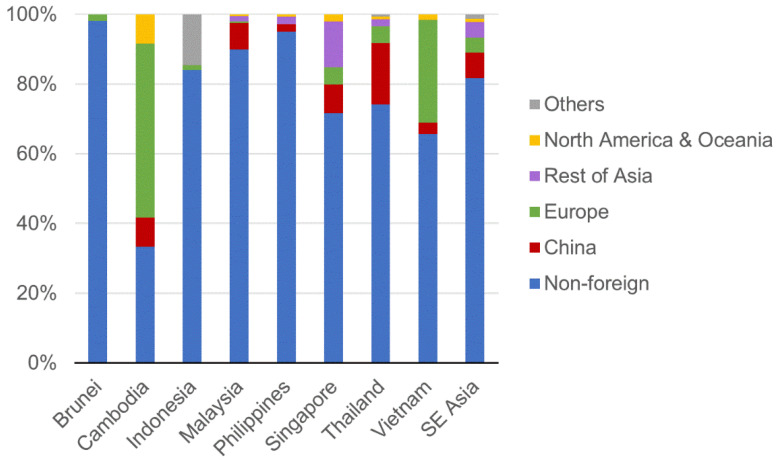
Nationality distribution of confirmed COVID-19 cases in Southeast Asia (SE Asia).

As shown in Figure 8, confirmed COVID-19 cases in Southeast Asia were predominantly local cases (81.1%). Cases among foreign nationals were chiefly from China (7.2%), Europe (5.0%), and other Asian countries (4.4%). Of all the countries investigated, Cambodia had the highest proportion of COVID-19 cases among foreign nationals (66.7%). However, when those with total case counts < 50 were excluded, Vietnam had the highest proportion of COVID-19 cases among foreign nationals (34.4%), whereas Brunei had the least (1.9%).

The median age of the 18 COVID-19-related deaths recorded during the study period was 58 years, which was significantly higher than that of surviving cases (43 years) (Mann-Whitney U Test; *p* < 0.01). Thirteen (72.2%) patients who died from COVID-19 infection had underlying conditions prior to the diagnosis of COVID-19. Of these, the most common underlying conditions were diabetes and/or chronic cardiovascular diseases (present in 10 cases). Dengue fever, asthma, and kidney transplantation were the underlying conditions present in the other three cases that died.

## 4. Discussion

This study retrospectively analyzed early population-level data for the COVID-19 outbreak in Southeast Asia. Relevant spatiotemporal patterns, demographic characteristics, and their heterogeneity among subregions were accessed for the first time. In addition, a mathematical model was successfully constructed to estimate the short-term incidence trends and indirectly to evaluate the effectiveness of early intervention strategies.

Epidemic curves and semi-logarithmic graphs consistently illustrated two distinct phases in the epidemic. The second phase began at the start of March 2020 and was characterized by a substantial increase in the number of reported cases. The sudden increase in confirmed COVID-19 cases was a consequence of mass gatherings for various events such as Sri Petaling tabligh (a Muslim religious gathering), which triggered cluster outbreaks in Malaysia [18]. It indicated that COVID-19 was entering a rapid transmission phase. WHO classified five countries in Southeast Asia (Indonesia, Malaysia, Singapore, Thailand, and Vietnam) as countries with local transmission on 2 March 2020 [19], and later declared the outbreak a pandemic on 11 March 2020 [2].

By comparing the temporal distribution characteristics of confirmed cases at the subregional level, the internal heterogeneity of the early diffusion of SARS-CoV-2 infection in Southeast Asia was revealed. Given the fact that the region is generally tropical, the uneven patterns of epidemic diffusion were likely to be determined by the diverse socio-demographic situations. The Human Development Index or HDI is a statistic composite that measures three key dimensions of human development: life expectancy, education, and per capita income [20]. On a global scale, the HDI was found to have a positive association with reported case counts in the early stages of COVID-19 pandemic [21,22]. According to the Human Development Report 2020, Singapore, Brunei, and Malaysia all have very high HDI, while the CLMV countries (Cambodia, Laos, Myanmar, and Vietnam) belonging to mainland Southeast Asia have the lowest within the region [23]. Therefore, our observations based on Southeast Asia supported HDI as an early predictor of reported COVID-19 diffusion. Learning from the experience of SARS in 2003, Singapore developed a hierarchical disease outbreak response system and accordingly took a timely and coordinated response to this COVID-19 outbreak [24]. Despite the disadvantages such as the extremely high population density and globalization level [21,25], a series of public health interventions adopted by Singapore in the early stage effectively slowed down the spread of the pandemic, which can be reflected in its relatively flat epidemic curve and the significantly lower ***R***_0_. The sharply rising epidemic curves and the abnormally high transmission dynamic estimates exposed some potential problems in the early responses of maritime Southeast Asian countries. As of mid-March 2020, Indonesia and the Philippines (with populations of over 100 million) both had extremely limited testing capacity which was less than 450 tests per day [26,27]. Undertesting can not only cover up the true severity of the pandemic, but also cause hidden dangers for the outbreaks in community settings.

Epidemics typically obey the law of exponential growth in their early stages, especially for infectious diseases with a ***R***_0_ > 1.0. Five studies reported ***R***_0_ estimates for the COVID-19 outbreak in China, with a range of 2.47–2.68 [28,29,30,31,32]. The overall ***R***_0_ of 2.51 in Southeast Asia fell in the same range, indicating a comparable transmissibility to that in China. Our study observed a similar exponential growth trend, which was applied to the prediction of a short-term incidence trend for COVID-19 within the region. The goodness-of-fit (adjusted R^2^) of the prediction model within the second phase of transmission was 0.96. Although the exponential growth model also fit the early epidemic patterns of COVID-19 in some other regions like Europe and Africa, the estimates of the relevant parameters in the model varied from each other [33,34]. This phenomenon could be related to different climatic conditions, genetic backgrounds, and sufficiency of health resources (especially detection capabilities). Our model predicted that the cumulative COVID-19 cases in Southeast Asia would exceed 10,000 by early April 2020. The cumulative number of COVID-19 patients in Southeast Asia was 10,153 as of 1 April 2020. This actual figure validates our prediction. Despite the effectiveness of short-term forecasts, we observed that actual case counts reported since Day 85 (6 April 2020) have gradually exceeded the lower limit of prediction. It is worth noting that Malaysia, which was the hardest hit in Southeast Asia at that time, implemented the Movement Control Order (MCO) nationwide on 18 March 2020 [35]. Thus, we speculate that the implementation of more stringent precautions may be a vital reason for the slowdown in cumulative case growth.

Geo-temporal maps illustrated the spatial distribution of early COVID-19 cases in Southeast Asia. Most of the confirmed cases were concentrated within several international metropolitan areas before spreading to smaller settlements. Many studies have demonstrated the link between transportation (especially air travel) and the spread of SARS-CoV-2 infection [36,37]. This seems to explain why metropolises, which are major international transportation hubs, experience higher exposure to outbreaks in the context of a pandemic. Diffusion theory in geography provides a conceptual framework for us to understand and summarize the spatial spread patterns of emerging infectious diseases. There are three basic types of diffusion: (1) spread outward from a source is called expansion diffusion, usually affecting adjacent spaces; (2) spread over a large distance is called relocation diffusion, often coming with migration, and (3) spread following a certain spatial hierarchy is called hierarchical diffusion [38]. Linka et al. identified the early spatial pattern of COVID-19 outbreak in Europe, which was characterized by relocation diffusion based on mobility networks [39]. Fortaleza et al. observed two patterns of early COVID-19 dispersion in Brazil: the expansion diffusion from the capital metropolitan area, and the hierarchical diffusion between São Paulo city and cities of regional relevance [40]. In this study, we found that the outbreak of COVID-19 in Southeast Asia, macroscopically, had experienced a spatial pattern shift, which was from relocation diffusion to hierarchical diffusion. This finding underscores the public health implications of travel restriction in reducing the COVID-19 pandemic. According to the spatiotemporal scanning, Malaysia as the center, along with Singapore, had formed the most likely spatiotemporal cluster of COVID-19 incidence in Southeast Asia since early March 2020, and the corresponding aggregation risk was 72.07 times that of the rest. This could be supported by the results of our previous phylogeographical study using genomic data [41]. Notably, the spread of the virus between neighboring countries is more in line with the spatial pattern of expansion diffusion.

Horizontal comparisons among different regions may lead to a better understanding of the early spatiotemporal patterns of this emerging infectious disease. On the African “Tropical Continent”, the COVID-19 outbreak was detected approximately one month later than that in Southeast Asia, and the first confirmed cases were primarily imported from Europe rather than mainland China [42]. This initial difference may be attributed to differentiated geo-relations and migration flows. Similar geographical variations in the early epidemic pattern could also be observed in the African region, highlighting that the burden of COVID-19 in North Africa was higher than that in sub-Saharan Africa [43], and the latter has a lower HDI level. Temporally, an abrupt exponential growth of African case counts emerged in the last week of March 2020 as the virus spread locally [43]. On the western edge of the Eurasian “World Island”, all 27 member states of the European Union (EU) were affected by COVID-19 within only 45 days [44]. Meanwhile, the outbreak dynamics of COVID-19 in Europe revealed multiple regional epicenters (involving France, Germany, Italy, and Spain) with exponential growth rates > 0.22/day [39,45]. Compared to Southeast Asia, Europe’s more rapid and extensive dispersal of COVID-19 was likely to be driven by deeper regional integration and looser travel restrictions.

We were not able to directly compare the distribution of age or sex within patient groups from different countries or regions because of considerable differences in population structure. However, we determined that the median age of non-foreign individuals with COVID-19 was significantly higher than that of their respective country’s general populations, which indicates that age may be a significant risk factor for COVID-19. This reinforces the previous finding that COVID-19 seems to be uncommon in children [46,47]. In terms of sex, the reported proportion of males in confirmed COVID-19 cases in China, Italy, and South Korea was 51.4%, 59.8%, and 37.7%, respectively [48,49,50]. Although more than half of the confirmed cases in Southeast Asia were male, the link between sex and COVID-19 susceptibility was not supported by the result of our corresponding hypothesis test. Moreover, participation in social activities may be considered an intermediary factor [46,50]. Interestingly, the age and sex composition of confirmed cases in Vietnam were unique in that many more young and female individuals were infected with COVID-19. This may be attributed to the population structure and the role of women in Vietnamese society. Nearly 20% of confirmed COVID-19 cases in Southeast Asia included foreign nationals, but this proportion varied between countries. Diversity in the composition of patients’ nationalities may suggest to some extent the risk of virus inputs. As an active center of the global community, frequent cross-border population movements increased human-to-human transmission within Southeast Asia [51]. Consequently, there is a need to give serious consideration to the rapid spread of the epidemic outside of China.

The global crude fatality rate for COVID-19 was 3.9% (6606 deaths out of 167,515 confirmed cases) as of 16 March 2020 (the end of our study period) [52]. The crude fatality rate for Southeast Asia was largely underestimated in this study because of delayed diagnosis and lack of transparency in the information given by health authorities. For example, the number of deaths from COVID-19 in Indonesia jumped from five on 16 March 2020 to 19 on 18 March 2020, thus increasing the crude fatality rate to 8.4% [53]. Although most COVID-19 patients may exhibit mild clinical symptoms, older people and individuals with underlying medical conditions may be at increased risk of suffering severe illness and death. Our study results are consistent with this finding [48,50]. The median age of the 18 deaths included in the study was significantly higher than that of surviving cases. Accordingly, the high fatality rate observed for early COVID-19 in the Philippines may be related to the relatively high median age of those infected. Of the COVID-19 patients who died, 72.2% had underlying conditions, such as diabetes and chronic cardiovascular diseases. Notably, one deceased case from Thailand also had dengue fever [54], a tropical disease that is common and active in Southeast Asia. It is difficult to distinguish these two viral diseases since they share some clinical and laboratory features. Public health security in this region is facing unprecedented challenges [55].

For those concerned with infection prevention and control policy, the historical epidemiology deepening the empirical record on past pandemics will be of particular value [56,57]. By mid-2021, a new wave of COVID-19 outbreaks associated with the Delta variant (B.1.617.2), which had originated in India, began to sweep through Southeast Asia [58]. A recent study demonstrated an exponential growth of daily new cases and a significant subregional heterogeneity in the lineage composition in Southeast Asia from March 2021 to June 2021 [59]. Regarding the COVID-19 burden, as of 20 July 2021, countries with a cumulative incidence of more than 1000 cases per 100,000 population in the region were among the maritime Southeast Asian countries (the highest was 2903 in Malaysia) [60]. Longitudinal comparisons reflected a degree of continuity in the spatiotemporal patterns of the COVID-19 pandemic in Southeast Asia. In addition, expansion diffusion seems to have an unignorable impact on the current spread of COVID-19, especially given the noteworthy increase in cross-border imported cases from Myanmar which is under political unrest [59,61]. Thus, travel restrictions (including border controls) remain to be necessary strategies to deal with the prevalence of emerging SARS-CoV-2 variants based on the les-sons from the early stages of the pandemic. Although several studies have confirmed the effectiveness of current vaccines against certain variants [62,63], the harsh reality is that vaccination coverage is far from the required herd immunity level in most parts of Southeast Asia [60]. Taking full account of the demographic risk factors that affect the susceptibility and severity of viral infections, prioritizing vulnerable populations such as the elderly could become a strategic choice in immunization programming.

Despite our efforts to ensure data quality and analytical rigor, the present study has several limitations. First, raw data were compiled from publicly available information, which was not equally available across the countries included in the study. Therefore, the data available for the overall analysis and sample size for demographic analysis were limited. Moreover, due to the delay in the diagnosis of COVID-19 infections and lack of transparency in the provided information, the number of COVID-19 cases and deaths may not have been comprehensively reported during the early phase of transmission. This may have resulted in an underestimation of the true severity of the outbreak in this region. Finally, evolving health policies and opportunistic factors make it difficult to predict pandemic trends. We used a simple and practical model to make short-term predictions of COVID-19 incidence trends in the study region.

## 5. Conclusions

This study was the first to describe the early epidemiological features and trends of the COVID-19 outbreak in Southeast Asia from a regional perspective. Analyses of spatiotemporal distribution characteristics indicated that the region’s COVID-19 situation was challenging and unevenly geographically distributed. At the national level, early disease burden was positively correlated with Human Development Index. Advanced age may play a significant role in increasing susceptibility to COVID-19 infection and can lead to severe clinical outcomes. Early transmission dynamics generally obeyed the law of exponential growth, whereas phase shifts subsequently occurred under the influence of interventions. Consequently, there is an urgent need to strengthen epidemic surveillance and improve resource allocation for combatting the pandemic.

## Figures and Tables

**Table 1:** Early estimation of epidemiological parameters for COVID-19 outbreak in Southeast Asia.

Region/Subregion	Cumulative Incidence ^1^ (per 1,000,000 Population)	Exponential Growth Rate ^2^ (per Day)	Doubling Time (Day)	Basic Reproduction Number, *R*_0_ (95% Confidence Interval)
Southeast Asia (SEA)	2.03	0.13	6.16	2.51 (2.31, 2.73)
Mainland SEA	0.90	0.08	8.22	3.14 (2.44, 3.97)
Singapore	41.87	0.06	6.81	1.37 (1.14, 1.63)
Maritime SEA	2.14	0.22	6.68	4.16 (3.75, 4.61)

^1^ As of 16 March 2020. ^2^ Exponential curve fitting for the second phase.

**Table 2:** Basic demographic characteristics of the study population.

Characteristic	Total (*n* = 925)	Mainland SEA (*n* = 220)	Singapore (*n* = 243)	Maritime SEA (*n* = 462)	*p*-Value ^1^
Age, year, median (IQR ^2^)	44(31–58)	37(29–51)	46(34–58)	46(32–59)	<0.001
Sex, *n* (%) ^3^ Male Female	514(55.6)	131(59.5)	140(57.6)	243(52.6)	0.188
	410(44.3)	89(40.5)	103(42.4)	218(47.2)	
Nationality, *n* (%)					<0.001
Foreign	168(18.2)	67(30.5)	69(28.4)	32(6.9)	
Non-foreign	750(81.1)	153(69.5)	174(71.6)	423(91.6)	
Unknown	7(0.7)	0	0	7(1.5)	

^1^ Kruskal-Wallis test (for age) or Chi-square test (for sex and nationality). ^2^ IQR = interquartile range. ^3^ Sex of one patient from maritime SEA was unknown.
